# Detection of the elusive Dwarf sperm whale (*Kogia sima*) using environmental DNA at Malpelo island (Eastern Pacific, Colombia)

**DOI:** 10.1002/ece3.7057

**Published:** 2021-03-04

**Authors:** Jean‐Baptiste Juhel, Virginie Marques, Andrea Polanco Fernández, Giomar H. Borrero‐Pérez, Maria Mutis Martinezguerra, Alice Valentini, Tony Dejean, Stéphanie Manel, Nicolas Loiseau, Laure Velez, Régis Hocdé, Tom B. Letessier, Eilísh Richards, Florine Hadjadj, Sandra Bessudo, Felipe Ladino, Camille Albouy, David Mouillot, Loïc Pellissier

**Affiliations:** ^1^ MARBEC University of Montpellier CNRS, Ifremer, IRD Montpellier France; ^2^ CEFE University of Montpellier CNRS EPHE‐PSL University IRD Univ Paul Valéry Montpellier 3 Montpellier France; ^3^ Instituto de Investigaciones Marinas y Costeras‐INVEMAR Museo de Historia Natural Marina de Colombia (MHNMC) Santa Marta Colombia; ^4^ SPYGEN Le Bourget‐du‐Lac France; ^5^ Institute of Zoology Zoological Society of London London UK; ^6^ Department of Environmental Systems Science Landscape Ecology Institute of Terrestrial Ecosystems ETH Universitӓt Zürich Zürich Switzerland; ^7^ Fundación Malpelo Bogotá Colombia; ^8^ IFREMER Unité Ecologie et Modèles pour l'Halieutique EMH Nantes France; ^9^ Unit of Land Change Science Swiss Federal Research Institute WSL Birmensdorf Switzerland

**Keywords:** eDNA, megafauna, mobile species, pelagic

## Abstract

1. Monitoring large marine mammals is challenging due to their low abundances in general, an ability to move over large distances and wide geographical range sizes.
2. The distribution of the pygmy (*Kogia breviceps*) and dwarf (*Kogia sima*) sperm whales is informed by relatively rare sightings, which does not permit accurate estimates of their distribution ranges. Hence, their conservation status has long remained Data Deficient (DD) in the Red list of the International Union for Conservation of Nature (IUCN), which prevent appropriate conservation measures.
3. Environmental DNA (eDNA) metabarcoding uses DNA traces left by organisms in their environments to detect the presence of targeted taxon, and is here proved to be useful to increase our knowledge on the distribution of rare but emblematic megafauna.
4. Retrieving eDNA from filtered surface water provides the first detection of the Dwarf sperm whale (*Kogia sima*) around the remote Malpelo island (Colombia).
5. Environmental DNA collected during oceanic missions can generate better knowledge on rare but emblematic animals even in regions that are generally well sampled for other taxa.

## Graphical abstract

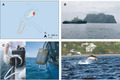

## 1 INTRODUCTION

Marine mammals are among the most threatened vertebrates on earth with 37% of them being considered as endangered by the IUCN (e.g., Albouy et al., 2020). Yet, the monitoring of marine mammals is still challenging, generally due to their low abundances, their ability to move over large distances, their wide geographical range sizes, and their elusive behavior (Hays et al., 2016). Most studies focusing on the distribution of relatively common marine animals rely on telemetry, passive acoustic surveys, or visual observations performed from the coast, during aerial surveys or during boat‐based surveys (e.g., Balmer et al., 2014; Mannocci et al., 2015; Palacios et al., 2012). By contrast, the distribution of rare or elusive mammal species are mainly investigated using compilations of scarce observations, fisheries bycatch and strandings (Coombs et al., 2019; Palacios et al., 2012; Plön, 2004). As a result, only a limited knowledge has been accumulated on the distribution of those species, which limits our capacity to set effective protection measures (Davidson et al., 2012). Developing complementary and effective tools for detecting and monitoring threatened, rare or elusive marine mammal species is key to better guide their conservation (Pikitch, 2018).

Environmental DNA (eDNA) metabarcoding is increasingly used to detect micro‐ and macro‐organisms in aquatic environments (Ruppert et al., 2019), but more case studies are needed to demonstrate its ability to detect unseen species that are elusive, threatened, and rare in marine ecosystems. The eDNA metabarcoding approach is based on retrieving DNA naturally released by organisms in their environment. This genetic material is then amplified by polymerase chain reaction (PCR), sequenced using high‐throughput DNA sequencing systems, and assigned to species based on a reference database (Taberlet et al., 2012). Most recent studies confirm the greater detectability of species using eDNA compared with traditional survey approaches in marine environments, especially those with a behavior that impede their direct observation (Boussarie et al., 2018; Pikitch, 2018; Simpfendorfer et al., 2016). For example, Thomsen et al. (2012) found eDNA to detect more species than nine conventional sampling methods of fish surveys in marine environments. Environmental DNA detection of cetaceans has been validated (Baker et al., 2018; Parsons et al., 2018) and can be used when direct observations are limited. For instance, the long‐finned pilot whale (*Globicephala melas*) was successfully detected in unexpected locations (Foote et al., 2012). The time sensitive nature of eDNA means that its detection is limited to a restricted area from where it was first shed and can be influenced by environmental factors such as currents and tides (Collins et al., 2018; Harrison et al., 2019).

**FIGURE 1:**
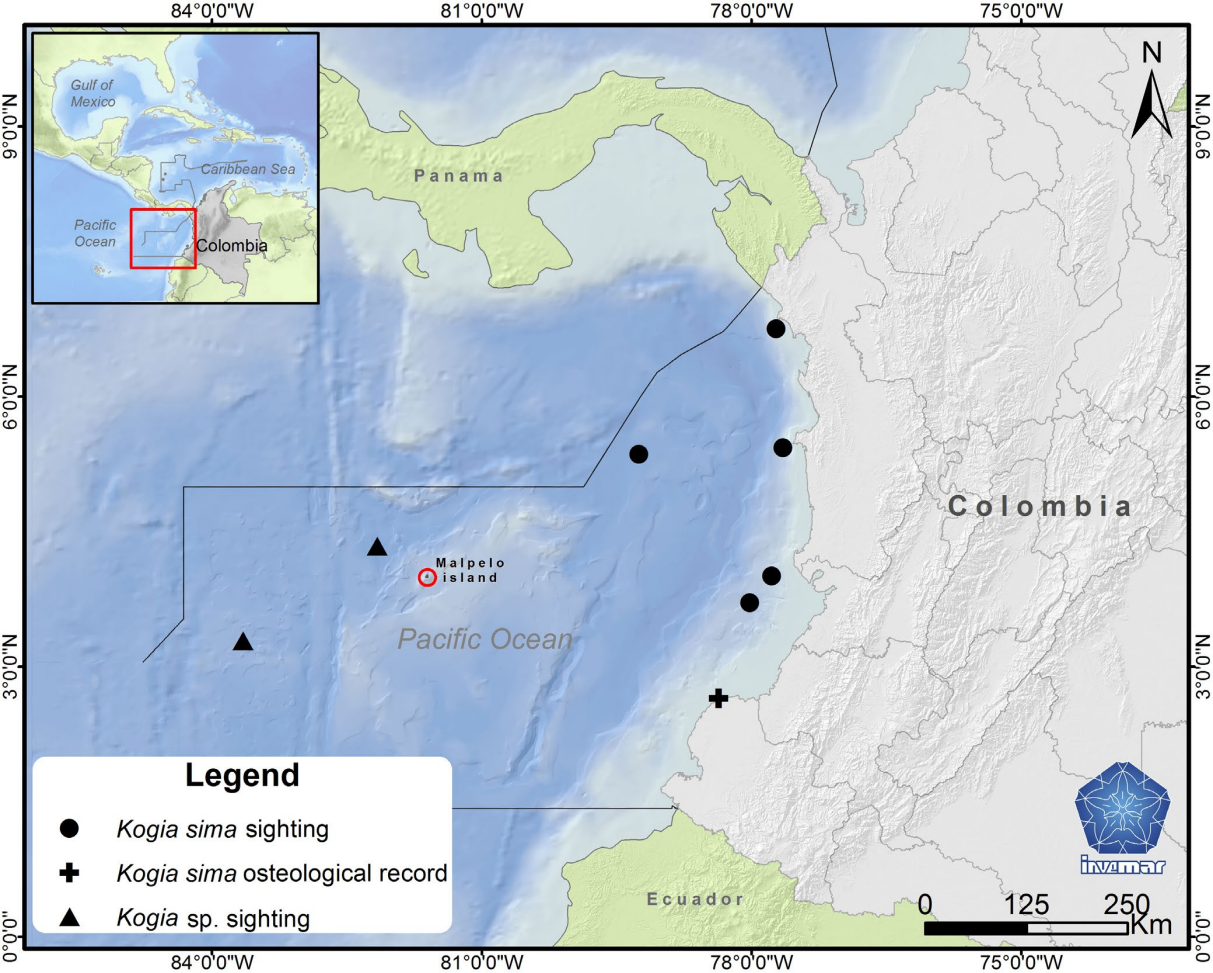
Map of Dwarf sperm whale (*Kogia sima*) et *Kogia sp*. sightings in the Colombian eastern Pacific

The pygmy (*Kogia breviceps*) and dwarf (*Kogia sima*) sperm whales are porpoise‐like shaped odontocetes smaller than 4 m (Plön, 2004) that are able to travel long distances (e.g., 255 nautical miles in 4 days, Scott et al., 2001). They occur worldwide in tropical and temperate waters including Colombia (Rice, 1998) and count 1,931 records (1,627 at the species level) of opportunistic sightings and strandings referenced in OBIS (Ocean Biogeographic Information System, www.obis.org, January 2020) and 2,503 records (2,223 at species level) in GBIF (Global Biodiversity Information Facility, www.gbif.org, for example, Mora‐Pinto et al., 1995). Their relatively scarce sightings prevent an accurate estimation of their distribution ranges and abundances while their conservation status has long remained Data Deficient (DD) in the Redlist of the IUCN. *Kogia sima* has been sighted only recently in the Colombian Caribbean (Mutis‐Martinezguerra et al., 2019) and only six occurrences have been documented in the Colombian Pacific, including five sightings and one stranding (Figure 1). Two sightings of *Kogia* sp. have been reported in the vicinity of the Malpelo volcanic island between 1986 and 2006 during line transect surveys, one was near the Island (Wade & Gerrodette, 1993) and the other one was 230 km WSW (Muñoz‐Hincapié et al., 1998; Palacios et al., 2012; Figure 1; Table 1). The Malpelo island (3°58’N, 81°37’W), covering an area of 1.2 km^2^, is located 490 km off the coast of Colombia, on the top of the submerged Malpelo ridge. This island is composed of barren rocks and steep edges with several underwater habitats including coral formations, vertical rock walls, sands and gravel, tunnels and caves. It is surrounded by deep waters with strong currents where at least nine cetacean species are present (Ávila et al., 2013; Herrera et al., 2007). These deep waters support important populations of large predators and pelagic species including giant grouper, billfish, short‐nosed ragged‐toothed shark, deepwater sharks and pelagic sharks (Unesco, 2005). Here we document the first detection of the uncommon Dwarf sperm whale (*Kogia sima*) around the remote Malpelo island (Colombia) using eDNA.

**TABLE 1:** Observation of *Kogia sima* et *Kogia* sp. in the Colombian Pacific

Species	Location			Author
	Lat	Long	Geographical reference	
*Kogia* sp.	3.291776°	−83.653121°	230 km WSW of Malpelo Island	Wade and Gerrodette (1993), Palacios et al. (2012)
*Kogia* sp.^a^	4.339118°	−82.159164°	Near Malpelo Island	Wade and Gerrodette (1993), Palacios et al. (2012)
*Kogia sima*	5.359326°	−79.247856°	225 km W off Cabo Corrientes	Muñoz‐Hincapié et al. (1998)
*Kogia sima*	6.753827°	−77.721361°	Near shore Cabo Marzo	Palacios et al. (2012)
*Kogia sima*	5.432542°	−77.647060°	Near shore Cabo Corrientes	Vidal (1990), Wade and Gerrodette (1993), Muñoz‐Hincapie et al. (1998), Palacios et al. (2012)
*Kogia sima*	4.008406°	−77.770061°	Off Bahía Málaga	Vidal (1990), Wade and Gerrodette (1993), Muñoz‐Hincapie et al. (1998), Palacios et al. (2012)
*Kogia sima*	3.713174°	−78.013709°	Off Bahía Málaga	Vidal (1990); Wade and Gerrodette (1993); Muñoz‐Hincapie et al. (1998), Palacios et al. (2012)
*)Kogia sima*	2.650000°	−78.360000°	Stranded animal between the communities of La Vigía and Mulatos	Muñoz‐Hincapie et al. (1998)

^a^ Confusing record assumed to be *Kogia sima* by Wade & Gerrodette, 1993.

## 2 METHODS

**FIGURE 2:**
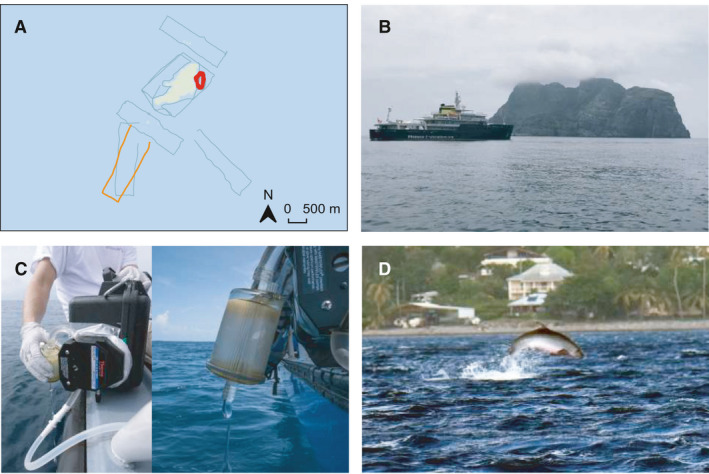
Location of *Kogia sima* detections around Malpelo Island using environmental DNA (red and orange track) (a); Malpelo Island seascape and our oceanographic vessel (b); eDNA filtration equipment (c) and Opportunistic sighting of *Kogia sp*. around Martinique (French West Indies) (d). *Kogia sima* was detected with both the Vert01 and Mamm01 primer pairs on the circular red track and detected with the Mamm01 primer pair on the rectangular orange track. It was not detected on the gray transects. Credit Photo R. Hocdé, C. Albouy, Megafauna project)

During an oceanographic expedition (March 2018, Figure 2a, b) seawater samples were collected in a 2 km radius around the island to investigate the marine vertebrate diversity. A total of 13 nonoverlapping 5 km‐long transects, either rectangular or circular, were performed. During each transect, duplicates of 30 L of subsurface seawater (between 0 and 40 cm) were simultaneously filtered using two peristaltic pumps placed on each side of the boat (Figure 2c) and two sterile filter capsules (VigiDNA 0.2 µm, SPYGEN). Immediately after, the filters were filled with conservation buffer (CL1 buffer, SPYGEN) and stored in the dark at ambient temperature. A contamination control protocol was carried out at both field and laboratory stages including the use of disposable gloves and single‐use filtration equipment (Goldberg et al., 2016; Valentini et al., 2016). The laboratory and equipment were not in contact with cetaceans or cetacean tissue, before or during the operations, and was cleaned with bleach before each sampling event and before each sample processing.

DNA extraction was performed in a dedicated eDNA laboratory equipped with separate clean rooms, positive air pressure, UV treatment and frequent air renewal. Decontamination procedures were conducted before and after all manipulations. Two extractions per filter were performed, following the protocol of Pont et al. (2018), and pooled after the amplification process. Two primer pairs were used for the amplification of metabarcoding sequences, a universal vertebrate 12S mitochondrial rDNA primer pair (Vert01, 5’‐TAGAACAGGCTCCTCTAG, 3’‐TTAGATACCCCACTATGC) and a mammal 12S mitochondrial rDNA primer pair (Mamm01, 5’ ‐CCGCCCGTCACYCTCCT, 3’‐GTAYRCTTACCWTGTTACGAC). Both were used with a human blocking primer pair (5’‐CTATGCTTAGCCCTAAACCTCAACAGTTAAATCAACAAAACTGCT ‐3’) (De Barba et al., 2014; Pont et al., 2018). The amplification primers were 5’‐labeled with an eight‐nucleotide tag unique to each sample (with at least three differences between any pair of tags), so all 12 PCRs from a single sample shared the same tag. The tags for the forward and reverse primers were identical for each sample. Twelve PCR replicates were run per filter. Three negative extraction controls and two negative PCR controls (ultrapure water) were amplified and sequenced in parallel to monitor possible contaminations. Two libraries were prepared using the MetaFast protocol (Fasteris, www.fasteris.com) and a paired‐end sequencing (2x125 bp) was carried out using an Illumina HiSeq 2,500 sequencer on two HiSeq Rapid Flow Cell v2 using the HiSeq Rapid SBS Kit v2 (Illumina, San Diego, CA, USA) following the manufacturer's instructions at Fasteris (Geneva, Switzerland). All sequences with a frequency of occurrence below 0.1% per taxon and library were discarded to avoid index cross‐talk (MacConaill et al., 2018) and tag‐jumps (Schnell et al., 2015). Additionally, sequences with less than 10 reads were removed. The metabarcoding workflow was based on the VSEARCH toolkit and the clustering algorithm SWARM that groups multiple sequence variants into OTUs (Operational Taxonomic Units) to clean errors from PCR and sequencing (Mahé et al., 2014; Rognes et al., 2016). The SWARM clustering algorithm uses single linkage clustering, in which sequence similarity and co‐occurrence patterns are used to group sequences together. It allows the removal of erroneous sequences and most reliable detections. Taxonomic assignment was performed using the ecotag program (lower common ancestor algorithm) from the OBITOOLS software package (Boyer et al., 2016) against the global and public EMBL genetic database (European Molecular Biology Laboratory, www.ebi.ac.uk, release 141 downloaded on 11th october 2019, Baker et al., 2000). Sequences assigned to common laboratory contaminants such as human, pig or dog were remove from analysis. Sequences were aligned using MUltiple Sequence Comparison by Log‐Expectation (MUSCLE) on MEGA software (www.megasoftware.net).

## 3 RESULTS

**FIGURE 3:**
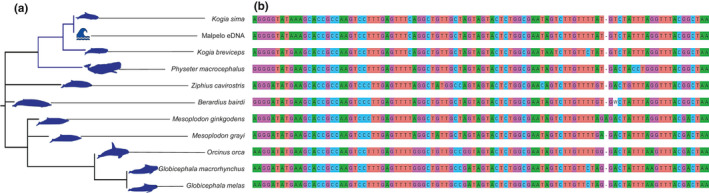
Phylogenetic tree of the toothed‐whales species (a) and their aligned sequences including the sequence from the eDNA sampling (b). The silhouettes were retrieved from phylopic.org (Chris Huh, Creative Commons Attribution‐ShareAlike 3.0 Unported)

From the 13 seawater samples, a total of 20,092,190 reads were produced with the vertebrate specific primer pair Vert01 and 4,321,072 reads with the mammal specific primer pair Mamm01. From these reads, 18,007,106 and 2,784,180 passed the bioinformatic cleaning process, respectively. Among the retained reads produced with the Vert01 primer pair, 431,758 reads were assigned to cetacean species including *Kogia sima*, *Grampus griseus* and two sequences of Delphinidae for which the primer was not resolutive. A total of 469 reads corresponding to a unique 99 bp sequence (Figure 3) matched at 100% similarity with the dwarf sperm whale 12S rDNA (*Kogia sima,* complete mitochondrial genome, Shan et al., 2019, NC_041303.1), at 97% similarity with the pygmy sperm whale (*Kogia breviceps*, Arnason et al., 2004, AJ554055.1), while only at < 96.4% with other phylogenetically close cetacean species referenced in EMBL (Gatesy et al., 2013; Figure 3). This sequence was detected on a single transect performed on the 25th of March 2018 at 17 PM (local time UTC −5, Figure 2a, circular transect).

Among the retained reads produced with the Mamm01 primer pair, 187,829 were identified to cetacean species including *Kogia sima*, *Globicephala macrorhynchus*, *Grampus griseus*, *Steno bredanensis* and two sequences of Delphinidae for which the primer was not resolutive. A total of 3,042 reads corresponding to a unique 63 bp sequence matched at 100% similarity with the same dwarf sperm whale 12S rDNA sequence and at 92.1% similarity with the pygmy sperm whale (*Kogia breviceps*, Arnason et al., 2004, AJ554055.1) referenced in EMBL. This sequence was detected on two transects performed on the 25th of March 2018 at 17 pm and the 27th of March 2018 at 10:30 a.m. (local time UTC −5, Figure 2a circular and rectangular transects) where 1,371 and 1,671 reads were respectively retrieved. Sea surface temperature, measured by the Naval Oceanographic Office (NAVOCEANO) and retrieved from the French Institute for Ocean Science repository (http://www.ifremer.fr/co-argoFloats/float?ptfCode=3901263) was 26.0°C and consistent with the thermal range of the dwarf sperm whale (10°C–30°C; www.obis.org).

## 4 DISCUSSION

Cetaceans include many threatened and difficult‐to‐study species for which eDNA is expected be a highly effective approach. Despite extensive efforts conducted over the span of 30 years, there are many gaps in the distribution records of those species (Figure 1). Environmental DNA metabarcoding can provide additional detections without visual observations (Boussarie et al., 2018). The two species *K. breviceps* and *K. sima* are very similar and very difficult to separate in the field leaving uncertain identifications in sighting records (Palacios et al., 2012). In contrast, environmental DNA can detect and identify accurately the species, avoiding observer related errors in records.

These results highlight the promises of eDNA as an alternative to standard monitoring methods for cetaceans, without requiring a close approach of a vessel. For example, Baker et al. (2018) show that eDNA of killer whales has been detected in seawater samples taken up to several hours after their passage and despite marine current circulation. Given its greater sensitivity and the fact that samples can be obtained from a wide variety of platforms (Harrison et al., 2019), eDNA has the potential to rapidly fill data gaps for cetaceans. Studies using this census method are usually limited by the completeness of genetic databases to taxonomically assign the retrieved sequences (Marques et al., 2020). However, strandings of cetaceans along the shores provide a valuable source of genetic material that can be sequenced on eDNA genetic markers to complete reference databases and investigate within species genetic diversity.

Opportunistic detections or targeted sampling in hotspots (e.g., Letessier et al., 2019) are expected to provide valuable new information on the occurrence of uncommon marine vertebrates and better define conservation plans. Malpelo island harbors a wide diversity of marine predators and presents all the characteristics of the last refuges for marine megafauna (Letessier et al., 2019). Thus, it deserves to be a priority for conservation and be placed under appropriate protection from human activities. Marine megafauna plays unique and irreplaceable functional roles in the ocean ecosystem such as the regulation of prey populations, removal of diseased individuals, transport of nutrients between habitats and over vast distances, and protection of blue carbon stocks (Atwood et al., 2015; Estes et al., 2016; Higgs et al., 2014).

Environmental DNA is a method that is easily applicable in the field and can benefit from the thousands of marine sampling operations that can take place regularly around the globe. These novel detections through eDNA will be crucial for Data Deficient species that can include a large proportion of threatened species (Bland et al., 2014; Parsons, 2016). Building on existing sampling efforts, filling reference database gaps and developing a large‐scale observatory network using environmental DNA from water collected in oceanic missions would contribute to a broader knowledge on those rare but emblematic animals.

## CONFLICT OF INTEREST

The authors declare no competing interest.

## AUTHOR CONTRIBUTION

**Jean‐Baptiste Juhel:** Conceptualization (lead); Data curation (equal); Formal analysis (supporting); Investigation (equal); Methodology (equal); Visualization (lead); Writing‐original draft (lead); Writing‐review & editing (equal). **Virginie Marques:** Data curation (equal); Formal analysis (lead); Software (lead); Writing‐review & editing (supporting). **Andrea Polanco Fernández:** Investigation (equal); Resources (equal). **Giomar H. Borrero‐Pérez:** Investigation (supporting); Writing‐review & editing (supporting). **Maria Mutis Martinezguerra:** Investigation (supporting); Writing‐review & editing (supporting). **Alice Valentini:** Formal analysis (equal); Software (equal); Writing‐review & editing (equal). **Tony Dejean:** Supervision (supporting); Writing‐review & editing (supporting). **Stephanie Manel:** Supervision (supporting); Writing‐review & editing (supporting). **Nicolas Loiseau:** Investigation (supporting). **Laure Velez:** Investigation (supporting). **Régis Hocdé:** Investigation (supporting). **Tom B. Letessier:** Investigation (supporting). **Eilísh Richards:** Visualization (supporting); Writing‐review & editing (supporting). **Florine Hadjadj:** Investigation (supporting). **Sandra Bessudo:** Validation (supporting). **Felipe Ladino:** Writing‐review & editing (supporting). **Camille Albouy:** Visualization (supporting); Writing‐review & editing (supporting). **David Mouillot:** Supervision (equal); Validation (equal); Writing‐review & editing (equal). **Loïc Pellissier:** Supervision (lead); Validation (equal); Writing‐review & editing (supporting).

## Data Availability

Code for the clustering bioinformatics pipeline can be found in Github: https://gitlab.mbb.univ-montp2.fr/edna/snakemake_rapidrun_swarm. The processed data that support the findings of this study are available in Dryad digital repository (https://doi.org/10.5061/dryad.66t1g1k0z).
